# An increased risk of pulmonary hypertension in patients with combined pulmonary fibrosis and emphysema: a meta-analysis

**DOI:** 10.1186/s12890-023-02425-4

**Published:** 2023-06-21

**Authors:** Hangqi Ni, Yuying Wei, Liuqing Yang, Qing Wang

**Affiliations:** Department of Respiratory Medicine, The First Affiliated Hospital, College of Medicine, Zhejiang University, 79 Qingchun Road, Hangzhou, Zhejiang 310003 People’s Republic of China

**Keywords:** CPFE, PH, IPF, COPD, Meta-analysis

## Abstract

**Background and aim:**

Pulmonary hypertension (PH) is a common complication of combined pulmonary fibrosis and emphysema (CPFE). Whether the incidence of PH is increased in CPFE compared with pure pulmonary fibrosis or emphysema remains unclear. This meta-analysis aimed to evaluate the risk of PH in patients with CPFE compared to those with IPF or COPD/emphysema.

**Methods:**

We searched the PubMed, Embase, Cochrane Library, and CNKI databases for relevant studies focusing on the incidence of PH in patients with CPFE and IPF or emphysema. Pooled odds ratios (ORs) and standard mean differences (SMD) with 95% confidence intervals (95% CIs) were used to evaluate the differences in the clinical characteristics presence and severity of PH between patients with CPFE, IPF, or emphysema. The survival impact of PH in patients with CPFE was assessed using hazard ratios (HRs).

**Results:**

A total of 13 eligible studies were included in the meta-analysis, involving 560, 720, and 316 patients with CPFE, IPF, and emphysema, respectively. Patients with CPFE had an increased PH risk with a higher frequency of pulmonary hypertension and higher estimated systolic pulmonary artery pressure (esPAP), compared with those with IPF (OR: 2.66; 95% CI: 1.55-4.57; *P* < 0.01; SMD: 0.86; 95% CI: 0.52-1.19; *P* < 0.01) or emphysema (OR: 3.19; 95% CI: 1.42-7.14; *P* < 0.01; SMD: 0.73; 95% CI: 0.50-0.96; *P* < 0.01). In addition, the patients with CPFE combined with PH had a poor prognosis than patients with CPFE without PH (HR: 6.16; 95% CI: 2.53–15.03; *P* < 0.01).

**Conclusions:**

Our meta-analysis showed that patients with CPFE were associated with a significantly higher risk of PH compared with those with IPF or emphysema alone. The presence of PH was a poor predictor of mortality.

**Supplementary Information:**

The online version contains supplementary material available at 10.1186/s12890-023-02425-4.

## Introduction

The concept of combined fibrosis and emphysema was first proposed by Mallory in 1984. It is characterized by predominant emphysema in the upper lobes and pulmonary fibrosis in the lower lobes [1]. The most common fibrotic pattern in combined pulmonary fibrosis and emphysema (CPFE) was UIP, and approximately half of CPFE-related studies required a diagnosis of IPF for patients with CPFE [2]. Besides, the presence of pulmonary emphysema was estimated to range from one quarter to one half in patients with IPF [3, 4]. However, the syndrome of CPFE with distinct clinical, functional, radiological, and prognostic characteristics has been acknowledged as a separate clinical entity by far [5].

Chronic pulmonary diseases, including obstructive pulmonary disease (COPD) and IPF, were associated with a high incidence of pulmonary hypertension (PH), and the patients with CPFE were particularly prone to the development of PH [6]. However, a number of studies have demonstrated a higher incidence of PH in patients with CPFE compared with those with IPF or emphysema [4, 7]. Some recent reports have shown no difference in PH presence between patients with CPFE and IPF or emphysema [8, 9]. Whether the cohabitation of pulmonary fibrosis and emphysema can raise the possibility of developing PH in patients with CPFE than in patients with IPF or emphysema alone remained unclear.

The presence of PH was associated with worse survival in patients with IPF and COPD [6]. Previous studies showed that PH was linked with an increased risk of death in patients with CPFE [10, 11]. However, it was also reported that the prognosis did not differ in patients with CPFE with or without PH [12]. The data on the effect of PH on survival in patients with CPFE were controversial.

The purpose of this study was to determine the risk of PH among patients with CPFE, IPF, or pure emphysema. In addition, we aimed to assess the effect of the presence of PH on mortality in patients with CPFE.

## Methods

### Literature search strategy

Our systematic meta-analysis was based on the PRISMA guidelines. Literature searches were conducted in electronic databases, including PubMed, Embase, CNKI, and Cochrane Library from inception to August 1, 2022, with the keywords “combined pulmonary fibrosis and emphysema” and “pulmonary hypertension.” The specific search strategies are illustrated in Supplementary File 1. We reviewed the retrieved studies and checked the full texts of potentially eligible studies.

### Selection criteria

The inclusion criteria for this study were as follows: (I) studies evaluating CPFE versus non-CPFE (IPF, emphysema, or COPD); (II) the diagnosis of CPFE as defined by Cottin et al. [10]; (III) PH confirmed by estimated systolic pulmonary arterial pressure (esPAP) evaluated using echocardiography; and (IV) data used to calculate odds ratio (OR) or standard mean difference (SMD) with 95% confidence interval (95% CI). The exclusion criteria were as follows: (I) case reports, conference abstracts, editorials, or reviews; (II) duplicate studies; and (III) studies with insufficient data.

### Data extraction and quality assessment

Two investigators (NHQ and LT) worked independently to extract data and assess the quality of the selected studies, with a third reviewer (WYY) to discuss and settle divergence. The following items were recorded: study characteristics (study design, duration, sample size, inclusion and exclusion criteria, and PH detection method), patient characteristics (age, sex, smoking state, and pulmonary function tests), and the presence of PH or esPAP. Newcastle–Ottawa Scale (NOS) was applied to assess the methodological quality of the eligible studies, with a score of 7 − 9 indicating high quality [13].

### Statistical analysis

The pooled OR and SMD were calculated to compare the possibility and severity of PH in patients with and without CPFE. Potential heterogeneity was measured using the *χ*^2^ test and *I*^2^ statistic. If the *I*^2^ was > 50%, the random-effects model was applied; otherwise, the fixed-effects model was adopted. The subgroup analysis was further performed to explore the source of heterogeneity.

Funnel plots were used as a form of qualitative analysis to evaluate the publication bias. A *P* value < 0.05 indicated a statistically significant difference. Statistical analysis was conducted using the R software (version 4.1.1).

## Results

### Study identification and selection

**Fig. 1:**
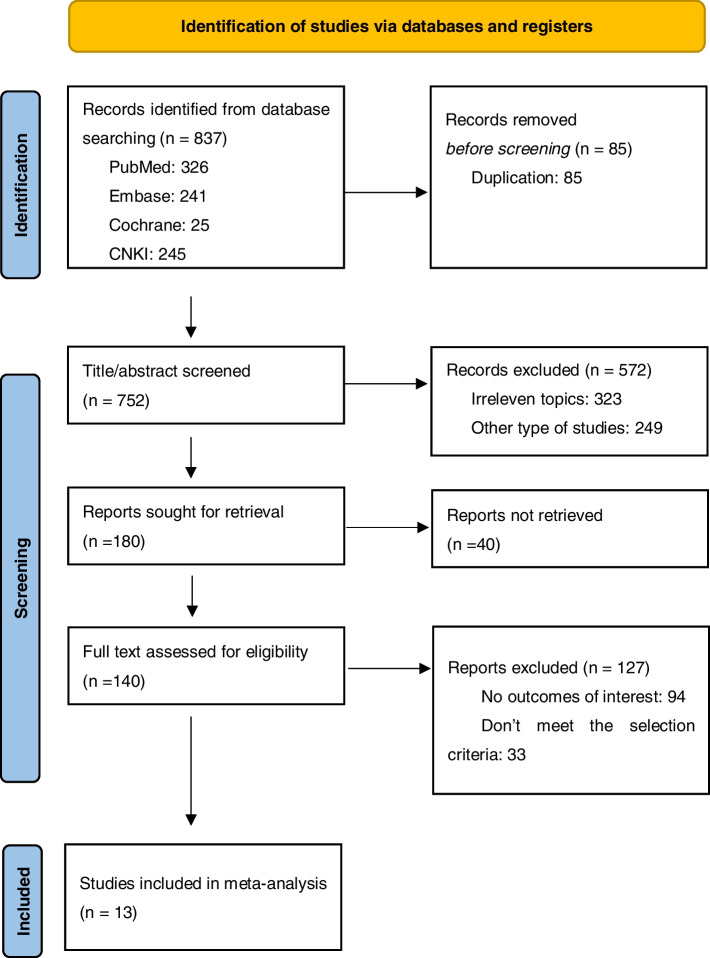
Flow diagram of the study selection process

The flow chart of the selection process is illustrated in Fig. 1. A total of 837 citations were obtained in the literature search. After removing 85 duplications, the remaining 752 studies were screened by titles and abstracts. Among these, 572 studies were removed because they were other types or irrelevant, and 40 studies were not retrieved. Then, full texts of 140 studies were further assessed for eligibility. Also, 127 publications were excluded due to a lack of interesting data or a mismatch with the selection criteria. Ultimately, 13 studies with 1596 participants were included in the meta-analysis, involving 560, 720, and 316 patients with CPFE, IPF, and emphysema, respectively [4, 7–9, 14–22].

### Characteristics of the included studies

The basic characteristics of the included studies are listed in Table 1. Studies published between 2009 and 2021 were mainly conducted in East Asia, apart from two in Turkey, one in Greece, one in Mexico, and one in the USA. Of the 16 studies, 8 studies compared patients with CPFE with patients with IPF, 3 studies compared patients with CPFE with patients with emphysema or COPD, and the remaining 2 studies analyzed all 3 groups.

**Table 1:** Basic characteristics of the selected studies in the meta-analysis

author/year	location	enrolled period	sample size			cutoff (mmHg)	NOS score
			**CPFE**	**IPF**	**COPD/E**		
Mejia, M./2009 [4]	Mexico	1996–2006	31	79		50	7
Lee, C. H./2011 [8]	China	2009–2012	135		135	35	8
Ryerson, C. J./2013 [14]	USA	2000.1–2010.6	29	336			7
Tzouvelekis, A./2013 [15]	Greece	2004.9–2010.8	40	60			6
He, L./2014 [7]	Korea	2001.1–2005.12	25	18	60		8
Chen, H./2014 [20]	China	2007.1–2010.1	31	36			7
Ye, Q./2014 [16]	China	2006.1–2009.12	70	55		50	7
Yang, N./2015 [21]	China	2012.12–2014.12	40		40		8
Chen, F./2016 [22]	China	2010.1–2015.2	21	26		40	8
Kohashi, Y./2016 [17]	Japan	2006.1–2012.12	8	39		45–50	8
Tomioka, H./2016 [18]	Japan	2007.5–2015.1	17		49		8
Ciftci, F./2019 [19]	Turkey	2013.9–2016.1	77	33			7
Ucsular, F./2021 [9]	Turkey	2013.9–2019.2	36	38	32	35	7

*CPFE* Combined pulmonary fibrosis and emphysema, *IPF* Idiopathic pulmonary fibrosis, *COPD/E* Chronic obstructive pulmonary diseases or emphysema only, *cutoff* cutoff on esPAP for diagnosis of PH, *NOS* Newcastle − Ottawa Scale

### Comparison of clinical parameters

We then explored the clinical differences between patients with and without CPFE. The results are summarized in Tables 2 and 3. The pooled data showed that patients with CPFE were associated with a male predominance (OR: 4.11; 95% CI: 1.92-8.80; *P* < 0.01), more ever-smokers (OR: 9.14; 95% CI: 3.51-23.75; *P* < 0.01), and longer smoking history (MD: 19.80; 95% CI: 12.63-26.97; *P* < 0.01) compared with patients with IPF. However, no significant differences were observed in sex, and number of smokers between patients with CPFE and those with emphysema.

**Table 2:** Meta-analysis on clinical characteristics of patients with CPFE compared to patients with IPF only

**Characteristic**	**Number of studies/patients**			**Pooled data**		**Heterogeneity**	
	**Study**	**CPFE**	**IPF**	**MD or OR [95% CI]**	***P***	**I**^2^** (%)**	**Ph**
Age and gender							
Age	9	328	660	-0.61 [-2.65, 1.43]	0.56	72	< 0.01
Gender (male)	10	368	720	4.11 [1.92, 8.80]	< 0.01	62	< 0.01
Smoking history							
Number of ever-smokers	8	266	669	9.14 [3.51, 23.75]	< 0.01	50	0.06
Pack years	8	262	440	19.80 [12.63, 26.97]	< 0.01	91	< 0.01
Pulmonary function test							
TLC, % of predicted	7	259	547	13.91 [10.58, 17.23]	< 0.01	55	0.04
FEV1/FVC, % of predicted	9	360	681	-6.95 [-9.49, -4.41]	< 0.01	79	< 0.01
DLCO, % of predicted	8	267	586	-10.05 [-15.04, -5.05]	< 0.01	84	< 0.01

*MD* Mean difference, *OR* Odds ratio, *95% CI* 95% confidence interval, *P P* value of pooled MD or OR; *I*^2^ value of *χ*2 based I-squared statistics, *Ph P* value of Heterogeneity test, *CPFE* Combined pulmonary fibrosis and emphysema, *IPF* Idiopathic pulmonary fibrosis, *TLC* Total lung capacity, *FVC/FEV1* Forced vital capacity/forced expiratory volume in 1 s, *DLCO* Diffusing capacity of the lung for carbon monoxide

**Table 3:** Meta-analysis on clinical characteristics of patients with CPFE compared to patients with emphysema only

**Characteristic**	**Number of studies/patients**			**Pooled data**		**Heterogeneity**	
	**Study**	**CPFE**	**COPD/E**	**MD or OR [95% CI]**	***P***	**I**^2^** (%)**	**Ph**
Age and gender							
Age	3	78	141	2.32 [0.14, 4.51]	0.04	42	0.18
Gender (male)	5	253	316	1.74 [0.92, 3.28]	0.09	0	0.42
Smoking history							
Number of ever-smokers	4	211	236	3.98 [0.69, 23.04]	0.12	50	0.14
Pack years	3	77	140	-9.06 [-14.77, -3.35]	< 0.01	0	0.44
Pulmonary function test							
TLC, % of predicted	2	61	92	-34.76 [-42.25, -27.26]	< 0.01	0	0.86
FEV1/FVC, % of predicted	3	78	141	24.91 [10.85, 38.96]	< 0.01	95	< 0.01
DLCO, % of predicted	2	61	92	-19.07 [-23.30. -14.84]	< 0.01	0	0.77

*MD* Mean difference, *OR* Odds ratio, *95% CI* 95% confidence interval, *P P* value of pooled MD or OR, *I2* value of χ2 based I-squared statistics, *Ph P* value of Heterogeneity test, *CPFE* Combined pulmonary fibrosis and emphysema, *COPD/E* Chronic obstructive pulmonary diseases or emphysema only, *TLC* Total lung capacity, *FVC/FEV1* Forced vital capacity/forced expiratory volume in 1 s, *DLCO* Diffusing capacity of the lung for carbon monoxide

With regard to the pulmonary function tests, the pooled data showed that DLCO was lower in patients with CPFE compared with those with IPF (MD: -10.05; 95% CI: -15.04 to -5.05; *P* < 0.01) and those with emphysema (MD: -19.07; 95% CI: -23.30 to -14.84; *P* < 0.01). Besides, patients with CPFE presented with a TLC higher than the TLC of patients with IPF (MD: 13.91; 95% CI: 10.58-17.23; *P* < 0.01) but lower than the TLC of patients with emphysema (MD: -34.76; 95%: -42.25 to -27.26; *P* < 0.01), and an FEV1/FVC lower than the FEV1/FVC of patients with IPF (MD: -6.95; 95% CI: -9.49 to -4.41; *P* < 0.01) but higher than the FEV1/FVC of patients with emphysema (MD: 24.91; 95% CI: 10.85-38.96; *P* < 0.01).

### PH risk in patients with CPFE versus patients with IPF

**Fig. 2:**
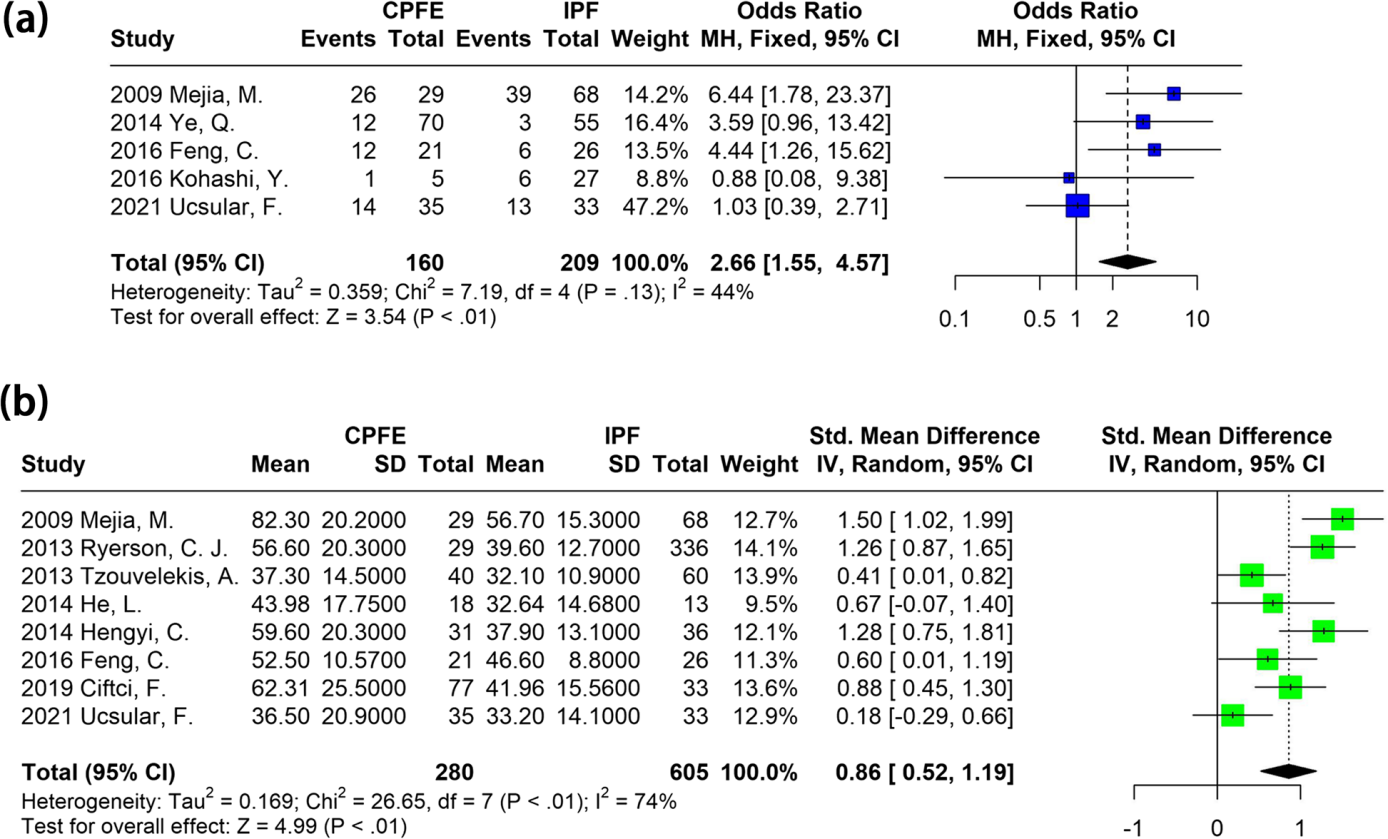
Meta-analysis of PH risk in patients with CPFE and IPF. **a** forest plot on relative risk of PH; **b** forest plot on SMD of esPAP

The results of the meta-analysis on the PH risk are presented in Fig. 2, comparing patients with CPFE and those with IPF. The probability of PH was higher in patients with CPFE than in patients with IPF (OR: 2.66; 95% CI: 1.55-4.57; *P* < 0.01). Besides, the pooled SMD of esPAP was also higher in patients with CPFE (SMD: 0.86; 95% CI: 0.52-1.19; *P* < 0.01).

**Fig. 3:**
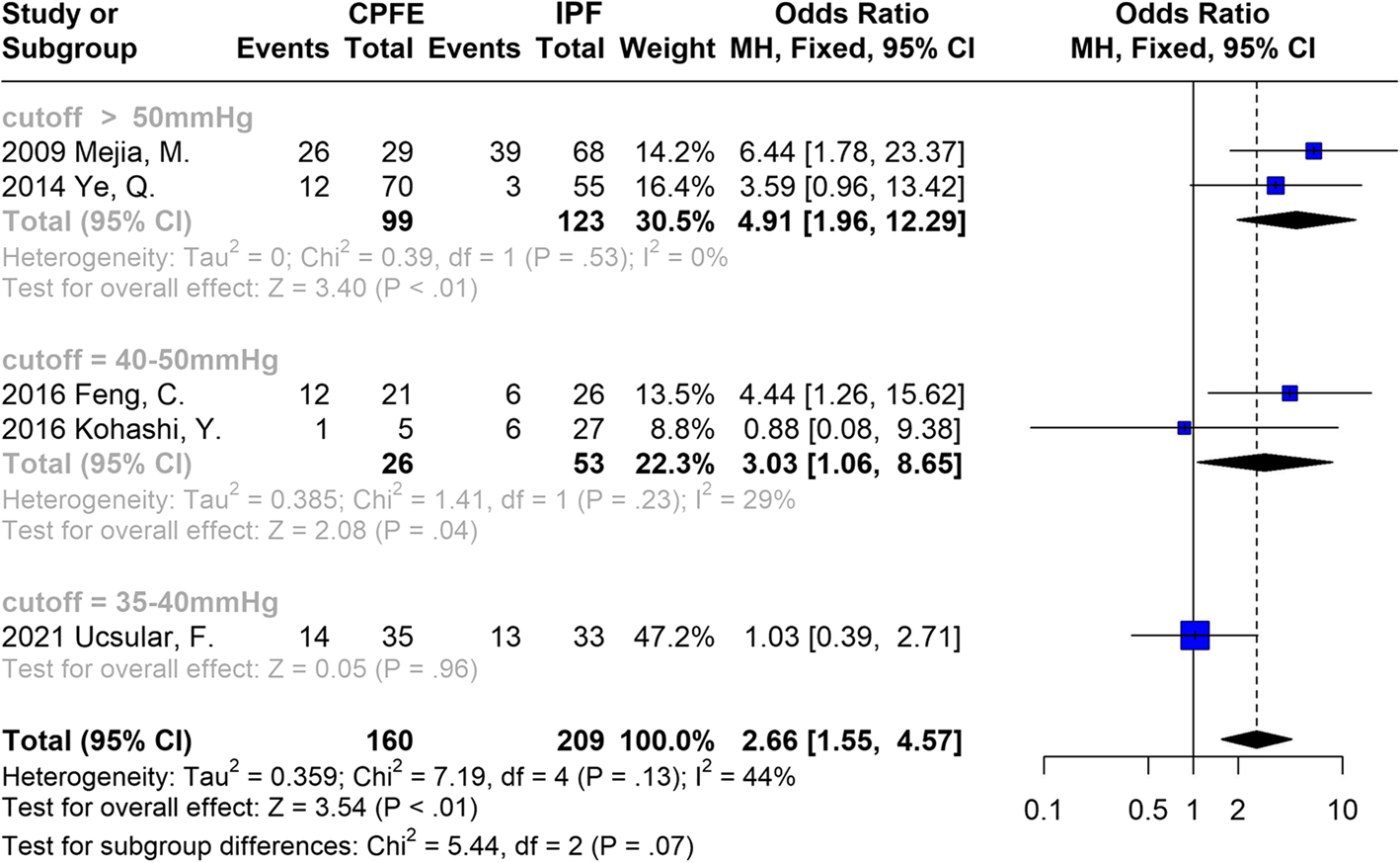
Subgroup meta-analysis of PH risk based on different thresholds of esPAP used for PH definition

Despite the low heterogeneity, subgroup analysis was performed according to the different thresholds of esPAP for PH diagnosis, as presented in Fig. 3. A significantly higher possibility of PH was observed in patients with CPFE with an esPAP cutoff > 50 mm Hg (OR: 4.91; 95% CI: 1.96–12.29; *P* < 0.01) and 40 − 50 mm Hg (OR: 3.03; 95% CI: 1.06-8.65; *P* < 0.05).

### PH risk in patients with CPFE versus patients with emphysema

**Fig. 4:**
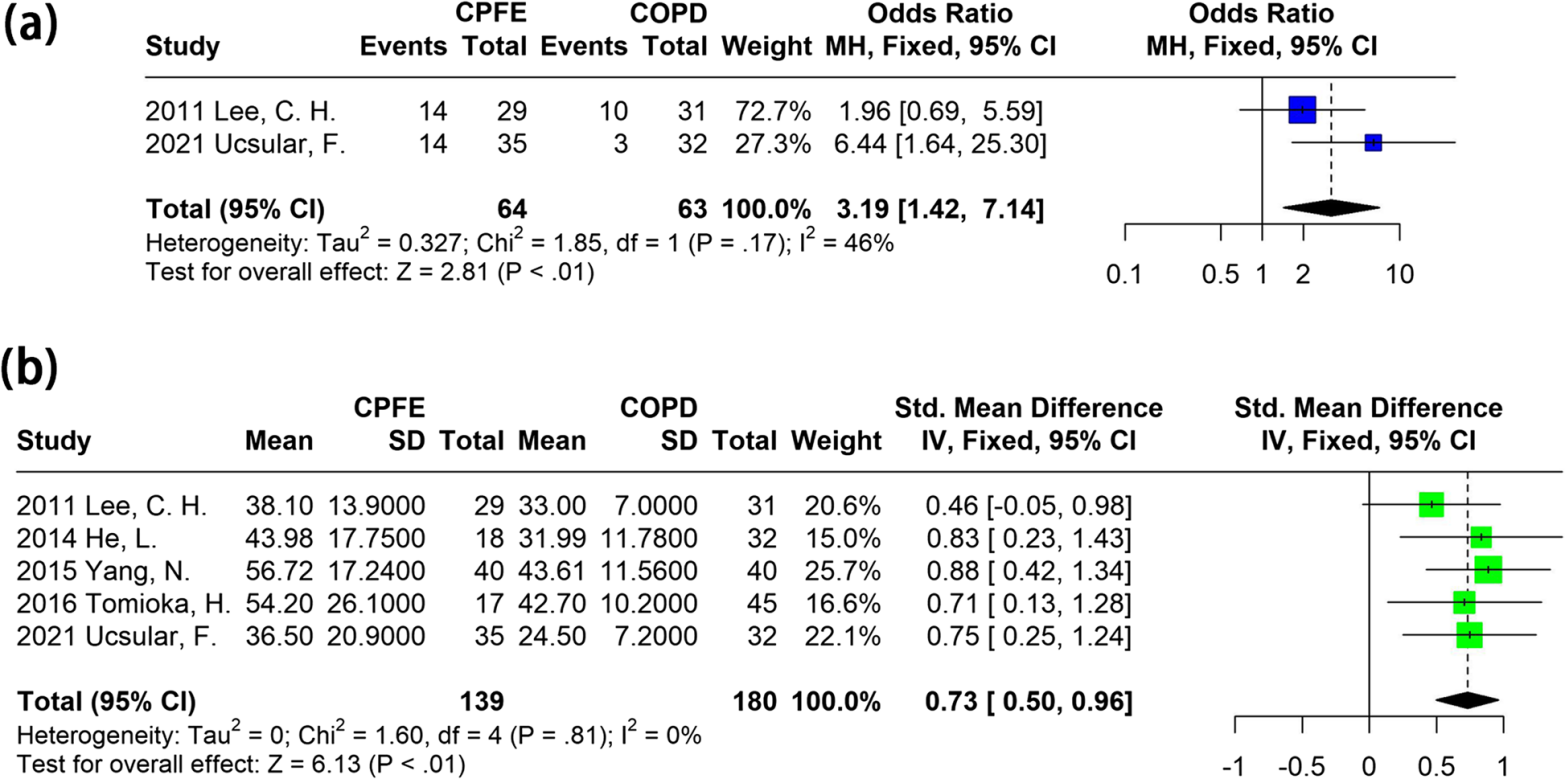
Meta-analysis of PH risk in patients with CPFE and emphysema only. **a** forest plot on relative risk of PH; **b** forest plot on SMD of esPAP

We further estimated the difference in the PH risk of patients with CPFE and those with pure pulmonary emphysema. As indicated by forest plots in Fig. 4, patients with CPFE had a higher incidence of PH compared with patients with emphysema only (OR: 3.19; 95% CI: 1.42-7.14; *P* < 0.01). In addition, the extent of PH indicated by esPAP was also higher in patients with CPFE (SMD: 0.73; 95% CI: 0.50-0.96; *P* < 0.01) than in those with emphysema alone.

### Effect of PH on survival in CPFE

**Fig. 5:**
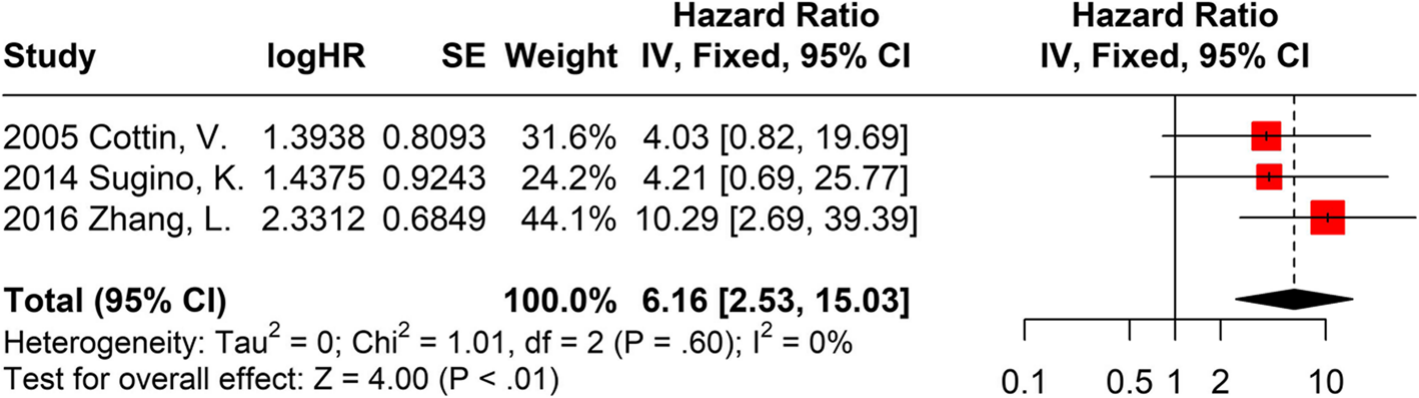
Meta-analysis of PH effect on survival

To disclose the effect of PH on survival in patients with CPFE, we recorded the HR of PH by repeating the selection and extraction process. The selection criteria and the analysis methods are shown in supplementary files (Supplementary Files 2, 3 and 4). The results of the synthesis are shown in Fig. 5. The overall analysis of three studies revealed that patients with CPFE with PH had a poorer overall survival (HR: 6.16; 95% CI: 2.53–15.03; *P*< 0.01) than patients with CPFE without PH [10, 12, 23].

### Sensitivity analysis and evaluation of publication bias

As the results of sensitivity analysis showed in the Supplementary File 5, the pooled data for PH risk did not change significantly after excluding any study, reflecting the stability of the meta-analysis.

**Fig. 6:**
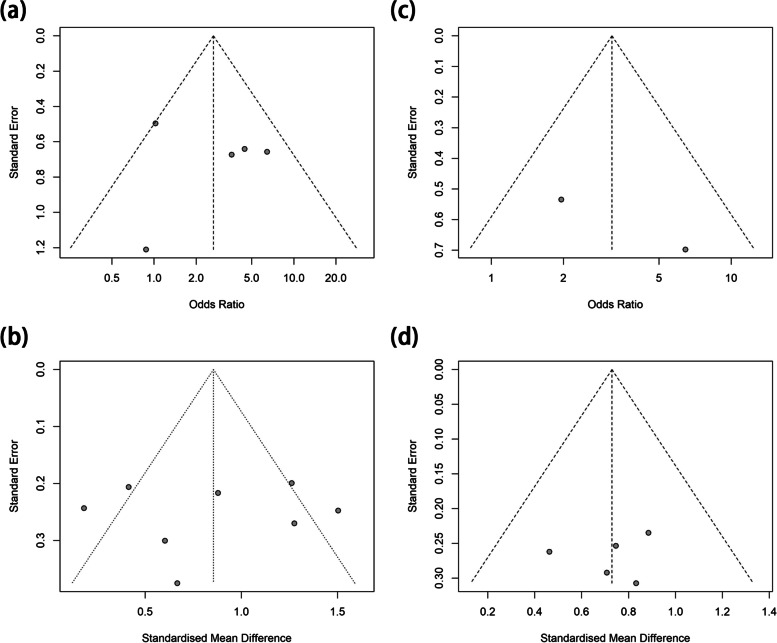
Funnel plot to assess evidence of publication bias. **a** funnel study for studies on PH risk between CPFE and IPF; **b** funnel study for studies on esPAP between CPFE and IPF. **c** funnel study for studies on PH risk between CPFE and emphysema; **d** funnel study for studies on esPAP between CPFE and emphysema

The publication bias was evaluated and the results were showed in Fig. 6. In the analysis involving the comparison of CPFE with IPF or emphysema, the funnel plots appeared to be symmetrical, indicating no evidence of publication bias.

## Discussion

The syndrome of CPFE has drawn considerable attention since it was first proposed, and has been regarded as a separate entity with distinct features. In accordance with previous studies, our meta-analysis showed that patients with CPFE had a larger male predominance and a greater exposure to cigarettes compared with those with IPF alone [4, 16]. However, no significant difference was observed in sex or smoking history in patients with CPFE than in patients with pure emphysema. It may be explained by the relatively small number of COPD patients which could have underestimated the differences.

It was reported that the combination of pulmonary fibrosis and emphysema resulted in different pulmonary function tests (PFT) in patients with either pure emphysema or pure IPF [7, 19]. IPF causes restrictive pulmonary physiology, whereas emphysema results in obstructive physiology. Consistent with previous studies, our meta-analysis showed a severely declined DLCO% in patients with CPFE, with a relatively preserved air flow rate and lung volume indicating a mix of restrictive and obstructive ventilatory defects and a more impaired diffusion capacity. It might partly explain the finding that patients with CPFE had a reduction in arterial oxygen partial pressure (PaO_2_) at rest and were more likely to manifest dyspnea and exercise limitation, as prior studies suggested [17, 24].

Secondary pulmonary arterial hypertension is defined as a mean pulmonary artery pressure (mPAP) ≥ 20 mm Hg, a pulmonary artery wedge pressure (PAWP) ≤ 15 mm Hg, and pulmonary vascular resistance (PVR) > 2 units at rest, evaluated by right heart catheterization (RHC) [25]. However, RHC is not routinely performed in clinical practice due to its invasiveness and high complication rates. Instead, echocardiography is applied as a regular noninvasive method [26]. In our meta-analysis, PH in included studies was estimated using systolic pulmonary artery pressure at echocardiography, with a different cutoff range from 35 to 50 mm Hg. The synthesis results indicated a statistically significant higher possibility of PH in patients with CPFE than in patients with IPF or emphysema alone. When we conducted the article search, we noted several studies using different methods to detect PH. Previous studies utilizing RHC to assess PH showed an increased risk of PH in patients with CPFE who exhibited higher mPAP or higher PVR than those with IPF [11, 27, 28]. Some studies found that patients with CPFE had higher RVSP, or more large pulmonary trunk size, which were hemodynamic and radiological signs of PH, respectively [29–31]. Additionally, in our meta-analysis, different cutoffs of esPAP to diagnose PH were used in the selected studies. Thus, a subgroup analysis was conducted, and the results demonstrated the highest risk of PH with esPAP thresholds ≥ 50 mm Hg, a higher risk with a cutoff between 40 and 50 mm Hg, and a non-significantly high risk with a cutoff between 35 and 40 mm Hg in patients with CPFE compared with patients with IPF alone. We speculate that patients with CPFE had a more significant hazard of PH when a stricter PH definition was used.

Several reasons might explain the increased risk of PH in patients with CPFE. First, it was assumed that the underlying parenchymal remodeling process caused some natural loss of overall vascular cross-sectional area and thus an increase in PVR [6]. Given the combined presence of fibrosis and emphysema, it was reasonable to contribute the high PH risk to the increased parenchymal abnormalities in patients with CPFE. Second, chronic hypoxia exposure could result in PH via multiple mechanisms, such as inducing inflammation, vasoconstriction, smooth muscle cell proliferation, and loss of distal pulmonary vessels [32]. Notably, the association between low DLCO and PH in patients with CPFE has been demonstrated in our current and previous studies [33, 34]. Therefore, it is reasonable to suspect that the higher likehood and greater severity of PH may be explained by the worse DLCO. This is supported by the clinical evidence that the severity of PH exhibits a negative correlation with both DLCO% and the volume of small pulmonary vessels [35–37]. Finally, an animal experimental using Ada-deficient mice successfully modeled both the features of CPFE, including airspace enlargement and fibrotic deposition, as well as PH, which is characterized by increased RVSP and vascular remodeling [38]. This study raised the possibility that the natural course of CPFE might encompass the development of PH. Additional studies focusing on the molecular mechanisms of PH in patients with CPFE are urgently required in the future.

With regard to histological vasculopathy in patients with CPFE, previous studies showed nonspecific, broad, and heterogeneous pulmonary vascular changes. The pathology involved the remodeling of arteries/arterioles, veins/venules, and capillaries, regardless of background lung parenchymal lesions. Compared with IPF or emphysema alone, the vascular changes and the Heath-Edwards grading (originally developed to assess pulmonary artery hypertension) were more severe in patients with CPFE. It was correlated with the increased risk of PH in patients with CPFE. Besides, the changes in the arteries/arterioles were the most obvious, suggesting that they played a major role in the development of PH. Plexiform lesions were rarely observed, and arteriopathy was not homogeneous, indicating that it did not belong to the vasculopathy type of idiopathic pulmonary arterial hypertension [34, 39].

Though the severity of PH and vascular remodeling was increased in patients with CPFE, it’s difficult to compare the association between PH with pulmonary emphysema or fibrosis. The prevalence of fibrosis and emphysema in PH-CLD was variable and contradictory across articles [36, 40]. Moreover, the correlation between the severity of PH and the scores of pulmonary emphysema or fibrosis remained unclear and incomparable [41–44]. In an attempt to shed more light on this topic, we conducted a meta-analysis of the 2 articles mentioned above with groups of IPF and COPD. The results revealed that the likelihood of PH wasn't different between patients with IPF and those with COPD, evidenced by an insignificantly increased OR of 2.83. However, the analysis was limited as we didn’t conduct comprehensive literature search on this topic and the extents of fibrosis and emphysema were not calculated. To date, it remains uncertain whether PH is more associated with pulmonary emphysema or fibrosis.

Our meta-analysis demonstrated that the presence of PH, evaluated mainly by echocardiography, was a significantly poor prognostic factor in the CPFE population. Previous studies using PVR or RVSP to assess PH also confirmed that the presence of PH had an adverse impact on survival in patients suffering from CPFE, with HR ranging from 1.01 to 10.29 [11, 45, 46]. Despite this dismal prognosis, there is currently no curative medication for the treatment of PH related to CPFE. Although several case reports indicated that the oral PH-specific therapies, such as ambrisentan, tadalafil, sitaxsentan and sildenafil, might improve hemodynamics, the potential clinical and survival benefits were unknow [47–49]. Controlled data do not support the use of oral PH-specific therapies, including endothelin receptor antagonists (bosentan, ambrisentan), phosphodiesterase-5 inhibitors (sildenafil, tadalafil), or stimulator of soluble guanylate cyclase (riociguat). Furthermore, treatment with ambrisentan and riociguat may be detrimental in patients with ILD and especially those with CPFE. To date, management of PH in the presence of CPFE is stilled based on managing the underlying respiratory disorder, treating hypoxemia with supplemental oxygen, and considering lung transplantation [1].

Although the presence of PH is a poor prognostic factor, the natural course of PH associated with CPFE remains to be fully explored. Generally, group 3 PH is chronic and progressive, but its course can be influenced by severity of underlying lung disease. For example, in IPF patients with mild-to-moderate restriction, the rate of the change of mPAP was 0.4 mm Hg/year [50]. But in advanced IPF patients who are transplant candidates, this rate increased to 3.8 mmHg/month [51]. Since patients with CPFE have both emphysema and fibross, it was convicing that the annual increase in esPAP was significantly greater in CPFE patients (11.3 ± 11.8 mmHg) than in those with IPF (2.4 ± 6.9 mmHg), as reported by Sugino et al. [23]

Our meta-analysis had several limitations. First, the official diagnostic criteria of CPFE were not established, and the diagnosis for CPFE usually included different types and extents of pulmonary fibrosis and emphysema in previous studies. Although we restricted the fibrosis pattern to IPF in the selected studies, the unstandardized criteria of CPFE might contribute to undue diagnosis or less sensitivity for patients with CPFE. Second, the emphysema group actually included patients diagnosed with COPD and patients without PFT, although they all had lung emphysema appearance on computed tomography. It could cause heterogeneity in the population with pulmonary emphysema. Third, most of the included studies had a retrospective collection of data, increasing the possibility that our analysis could be subject to selection bias. Finally, it was possible that the inclusion of studies with a small size of the population might have resulted in statistical bias.

In conclusion, our meta-analysis revealed a significantly increased risk of PH in patients with CPFE compared with those with IPF or emphysema alone. Also, the presence of PH was a significant predictor of mortality. However, due to the limitations in this study, multi-center or prospective studies with a clear definition of CPFE should be conducted in the future to further explore the risk of PH and its impact on survival in patients with CPFE.

## Supplementary Information

**Additional file 1: Supplementary Table 1.** Search criterion for the analysis of the risk of PH in patients with CPFE.

**Additional file 2: Supplementary Table 2.** Selecting criterion for the analysis of the effect of PH on survival in CPFE patients.

**Additional file 3: Supplementary File 3.** Flow diagram for the analysis of the effect of PH on survival in CPFE patients.

**Additional file 4: Supplementary Table 4.** Basic characteristics in analysis of the effect of PH on survival in CPFE patients.

**Additional file 5: Supplementary Figure 1. **Sensitivity Analysis of combined OR between CPFE patients and IPF.

## Data Availability

The data analyzed during this study are included in the published articles and its supplementary information.

## Abbreviations

PH: Pulmonary hypertension
CPFE: Combined pulmonary fibrosis and emphysema
COPD: Obstructive pulmonary disease
IPF: Idiopathic pulmonary fibrosis
UIP: Usual interstitial pneumonitis
CNKI: China national knowledge infrastructure
esPAP: Estimated systolic pulmonary artery pressure
RVSP: Right ventricular systolic pressure
RHC: Right heart catheterization
ORs: odds ratios
SMD: Standard mean differences
95% CIs: 95% Confidence intervals
HRs: Hazard ratios
TLC: Total lung capacity
FVC/FEV1: Forced vital capacity/forced expiratory volume in 1 s
DLCO: Diffusing capacity of the lung for carbon monoxide
