# Supplementary material for: An increased risk of pulmonary hypertension in patients with combined pulmonary fibrosis and emphysema: a meta-analysis
Source: BMC Pulm Med. 2023 Jun 21;23:221. doi: 10.1186/s12890-023-02425-4 (PMC10283193; doi:10.1186/s12890-023-02425-4)
Supplement: Supplementary file 1 — Additional file 1: Supplementary Table 1. Search criterion for the analysis of the risk of PH in patients with CPFE. [file 12890_2023_2425_MOESM1_ESM.docx]

##### Supplementary Table 1. Search criterion for the analysis of the risk of PH on patients with CPFE.

##### 1a. Search criterion of Medline (August 1st, 2022) (n=326).

| Search  NO. | Query Results | Items found |
| --- | --- | --- |
| #1 | "CPFE"[All Fields] OR (("combinable"[All Fields] OR "combinated"[All Fields] OR "combination"[All Fields] OR "combinational"[All Fields] OR "combinations"[All Fields] OR "combinative"[All Fields] OR "combine"[All Fields] OR "combined"[All Fields] OR "combines"[All Fields] OR "combining"[All Fields]) AND ("pulmonary fibrosis"[MeSH Terms] OR ("pulmonary"[All Fields] AND "fibrosis"[All Fields]) OR "pulmonary fibrosis"[All Fields]) AND ("emphysema"[MeSH Terms] OR "emphysema"[All Fields] OR "emphysemas"[All Fields] OR "pulmonary emphysema"[MeSH Terms] OR ("pulmonary"[All Fields] AND "emphysema"[All Fields]) OR "pulmonary emphysema"[All Fields])) | 666 |
| #2 | "pulmonary arterial hypertension"[MeSH Terms] OR ("pulmonary"[All Fields] AND "arterial"[All Fields] AND "hypertension"[All Fields]) OR "pulmonary arterial hypertension"[All Fields] OR "PAH"[All Fields] OR ("hypertension, pulmonary"[MeSH Terms] OR ("hypertension"[All Fields] AND "pulmonary"[All Fields]) OR "pulmonary hypertension"[All Fields] OR ("pulmonary"[All Fields] AND "hypertension"[All Fields])) OR ("physiology"[MeSH Subheading] OR "physiology"[All Fields] OR "ph"[All Fields]) OR "pulmonary arterial hypertension"[MeSH Terms] | 13610295 |
| #8 | #1 AND #2 | 326 |

##### 1b. Search criterion of Embase (August 1, 2022) (n=241).

| Search  NO. | Query Results | Items found |
| --- | --- | --- |
| #1 | cpfe OR (combined AND pulmonary AND fibrosis AND emphysema) | 1000 |
| #2 | 'combined pulmonary fibrosis and emphysema'/exp | 72 |
| #3 | 'combined pulmonary fibrosis and emphysema syndrome'/exp | 14 |
| #4 | 'pulmonary hypertension' | 112964 |
| #5 | 'pulmonary hypertension'/exp | 112086 |
| #6 | #1 OR #2 OR #3 | 1000 |
| #7 | #4 OR #5 | 121723 |
| #8 | #6 AND #7 | 241 |

##### 1c. Search criterion of Cochrane Library (August 1st, 2022) (n=25).

| Search  NO. | Query Results | Items found |
| --- | --- | --- |
| #1 | (CPFE):ti,ab,kw OR (combined pulmonary fibrosis and emphysema):ti,ab,kw OR (CPFE) OR (combined pulmonary fibrosis and emphysema) (Word variations have been searched) | 80 |
| #2 | ("pulmonary arterial hypertension") OR (PAH) OR ("pulmonary hypertension") OR ("pulmonary hypertension syndrome") OR (PH) | 29196 |
| #3 | MeSH descriptor: [Pulmonary Arterial Hypertension] explode all trees | 47 |
| #4 | MeSH descriptor: [Hypertension, Pulmonary] explode all trees | 1296 |
| #5 | #2 OR #3 OR #4 | 29299 |
| #6 | #1 AND #5 | 25 |

##### 1d. Search criterion of CNKI (August 1st, 2022) (n=245).

FT = （‘combined pulmonary fibrosis and emphysema’ + ‘CPFE’）AND FT=（‘pulmonary hypertension’）
