# Supplementary material for: An increased risk of pulmonary hypertension in patients with combined pulmonary fibrosis and emphysema: a meta-analysis
Source: BMC Pulm Med. 2023 Jun 21;23:221. doi: 10.1186/s12890-023-02425-4 (PMC10283193; doi:10.1186/s12890-023-02425-4)
Supplement: Supplementary file 2 — Additional file 2: Supplementary Table 2. Selecting criterion for the analysis of the effect of PH on survival in CPFE patients. [file 12890_2023_2425_MOESM2_ESM.docx]

##### Supplementary table 2. Selecting criterion for the analysis of the effect of PH on survival in CPFE patients.

| The inclusion criteria |
| --- |
| 1. studies evaluated CPFE with PH versus CPFE without PH; 2. the diagnosis of CPFE was defined by Cottin et al; 3. PH was confirmed by estimated systolic pulmonary arterial pressure (esPAP) evaluated by echocardiography or pulmonary vascular resistance (PVR) evaluated by right heart catheterization; 4. Data was capable to collect hazard ratio (HR) and 95% confidence interval (95%CI). |
| The exclusion criteria |
| 1. case reports, conference abstracts, editorials, or reviews; 2. duplicate studies; 3. articles with insufficient data. |
