# Supplementary material for: An increased risk of pulmonary hypertension in patients with combined pulmonary fibrosis and emphysema: a meta-analysis
Source: BMC Pulm Med. 2023 Jun 21;23:221. doi: 10.1186/s12890-023-02425-4 (PMC10283193; doi:10.1186/s12890-023-02425-4)
Supplement: Supplementary file 3 — Additional file 3: Supplementary File 3. Flow diagram for the analysis of the effect of PH on survival in CPFE patients. [file 12890_2023_2425_MOESM3_ESM.docx]

##### Supplementary file 3. Flow diagram for the analysis of the effect of PH on survival in CPFE patients.

**Identification of studies via databases and registers**

Records removed

*before screening* (n = 85)

Duplication: 85

Records identified from database searching (n = 837)

PubMed: 326

Embase: 241

Cochrane: 25

CNKI: 245

**Identification**

Records excluded (n = 540)

Irreleven topics: 323

Other type of studies: 217

(n = )

Title/abstract screened

(n = 752)

Reports sought for retrieval

(n =212)

Reports not retrieved

(n =66)

**Screening**

Reports excluded (n = 142)

No outcomes of interest: 139

Don’t meet the selection criteria: 4

Full text assessed for eligibility

(n =146)

Studies included in meta-analysis

(n = 3)

**Included**
