# Supplementary material for: An increased risk of pulmonary hypertension in patients with combined pulmonary fibrosis and emphysema: a meta-analysis
Source: BMC Pulm Med. 2023 Jun 21;23:221. doi: 10.1186/s12890-023-02425-4 (PMC10283193; doi:10.1186/s12890-023-02425-4)
Supplement: Supplementary file 4 — Additional file 4: Supplementary Table 4. Basic characteristics in analysis of the effect of PH on survival in CPFE patients. [file 12890_2023_2425_MOESM4_ESM.docx]

##### Supplementary table 4. Basic characteristics in analysis of the effect of PH on survival in CPFE patients.

| **author/year** | **location** | **enrolled period** | **type** | **sample size** | | **cutoff** | **NOS score** |
| --- | --- | --- | --- | --- | --- | --- | --- |
|  |  |  |  | **PH** | **non-PH** |  |  |
| Cottin, V./2005 | France | 1985.1 - 2003.12 | retrospective | 20 | 23 | 45mmHg | 6 |
| Sugino, K./2014 | Japan | 2003.4 - 2010.12 | retrospective | 16 | 9 | 30.5mmHg | 6 |
| Zhang, L./2016 | China | 2001.1 - 2013.12 | retrospective | 39 | 48 | RHC* | 6 |

*: a pulmonary artery wedge pressure (PAWP) ≤15 mm Hg, and pulmonary vascular resistance (PVR) >3 units at rest.
