# Supplementary material for: An increased risk of pulmonary hypertension in patients with combined pulmonary fibrosis and emphysema: a meta-analysis
Source: BMC Pulm Med. 2023 Jun 21;23:221. doi: 10.1186/s12890-023-02425-4 (PMC10283193; doi:10.1186/s12890-023-02425-4)
Supplement: Supplementary file 5 — Additional file 5: Supplementary Figure 1. Sensitivity Analysis of combined OR between CPFE patients and IPF. [file 12890_2023_2425_MOESM5_ESM.pptx]

## Slide 1
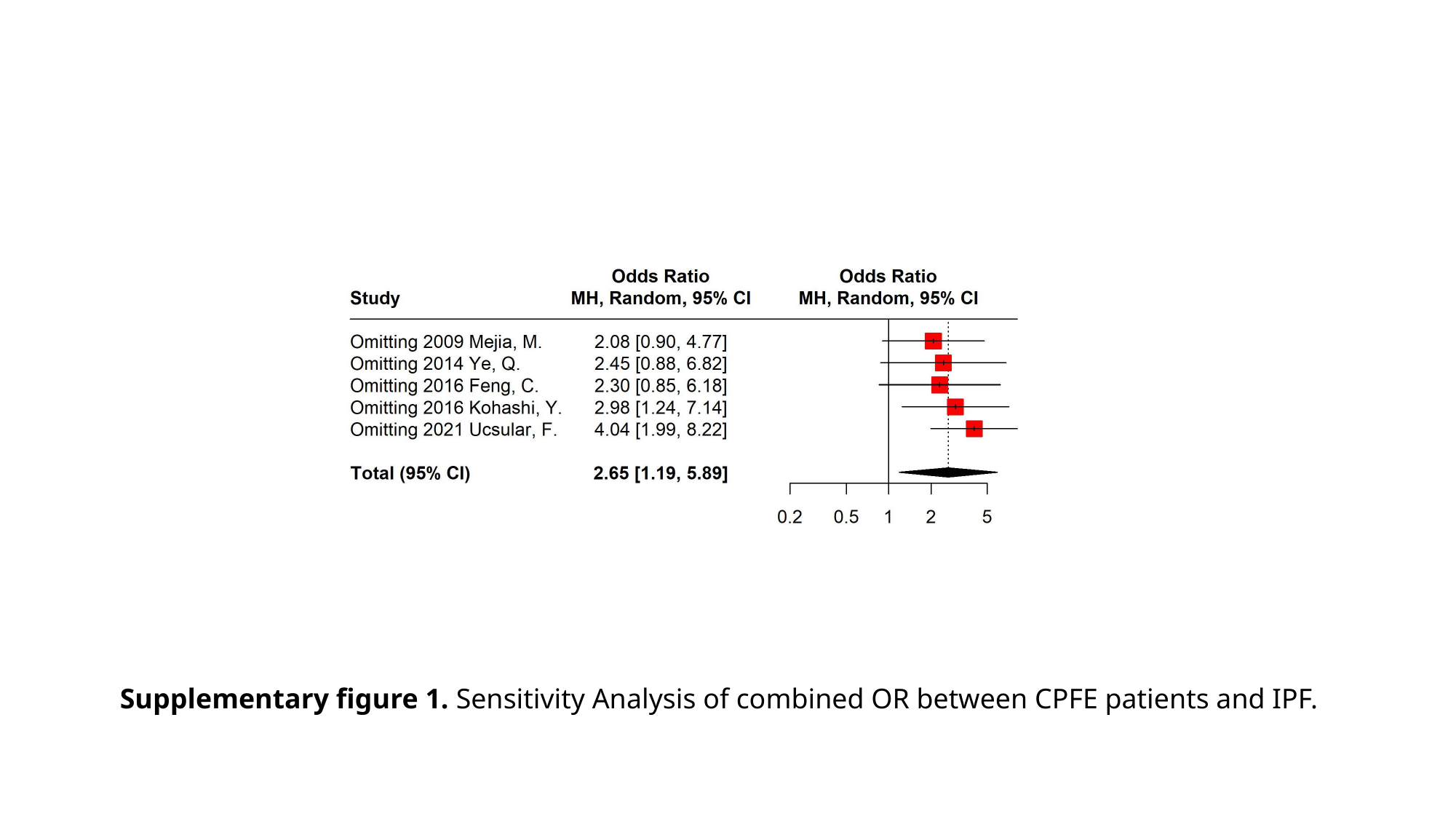

Supplementary figure 1. Sensitivity Analysis of combined OR between CPFE patients and IPF.

## Slide 2
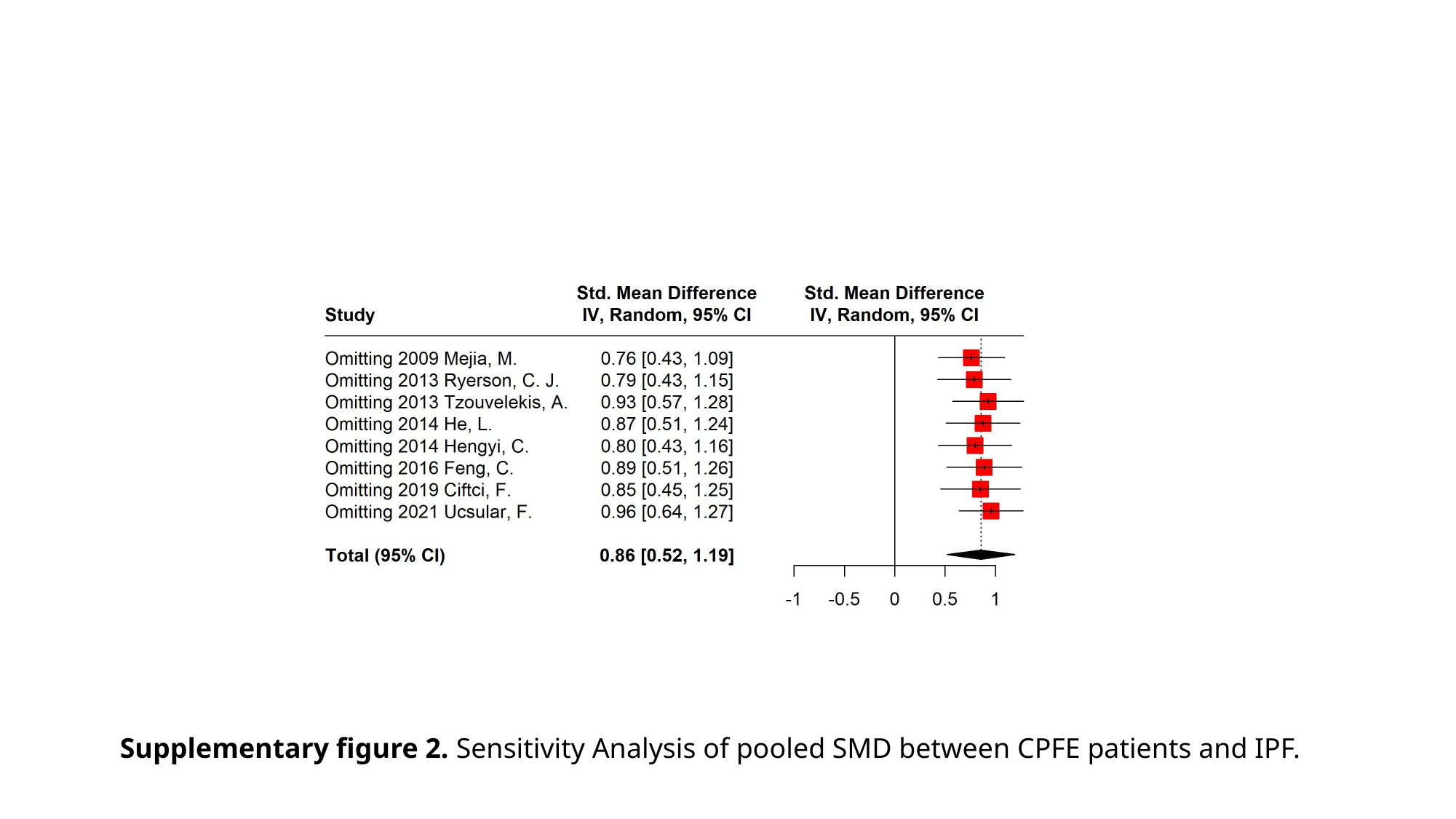

Supplementary figure 2. Sensitivity Analysis of pooled SMD between CPFE patients and IPF.

## Slide 3
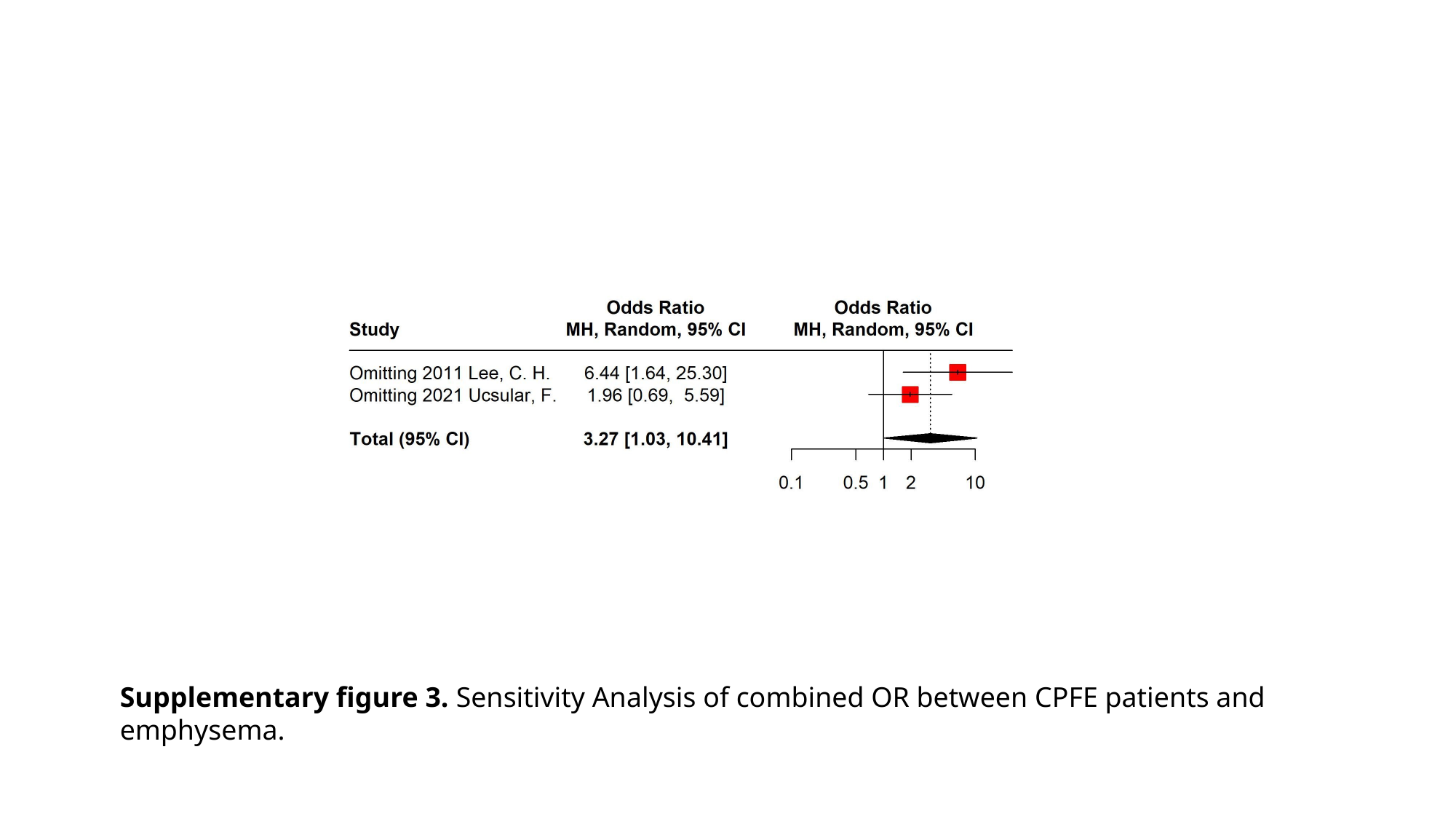

Supplementary figure 3. Sensitivity Analysis of combined OR between CPFE patients and emphysema.

## Slide 4
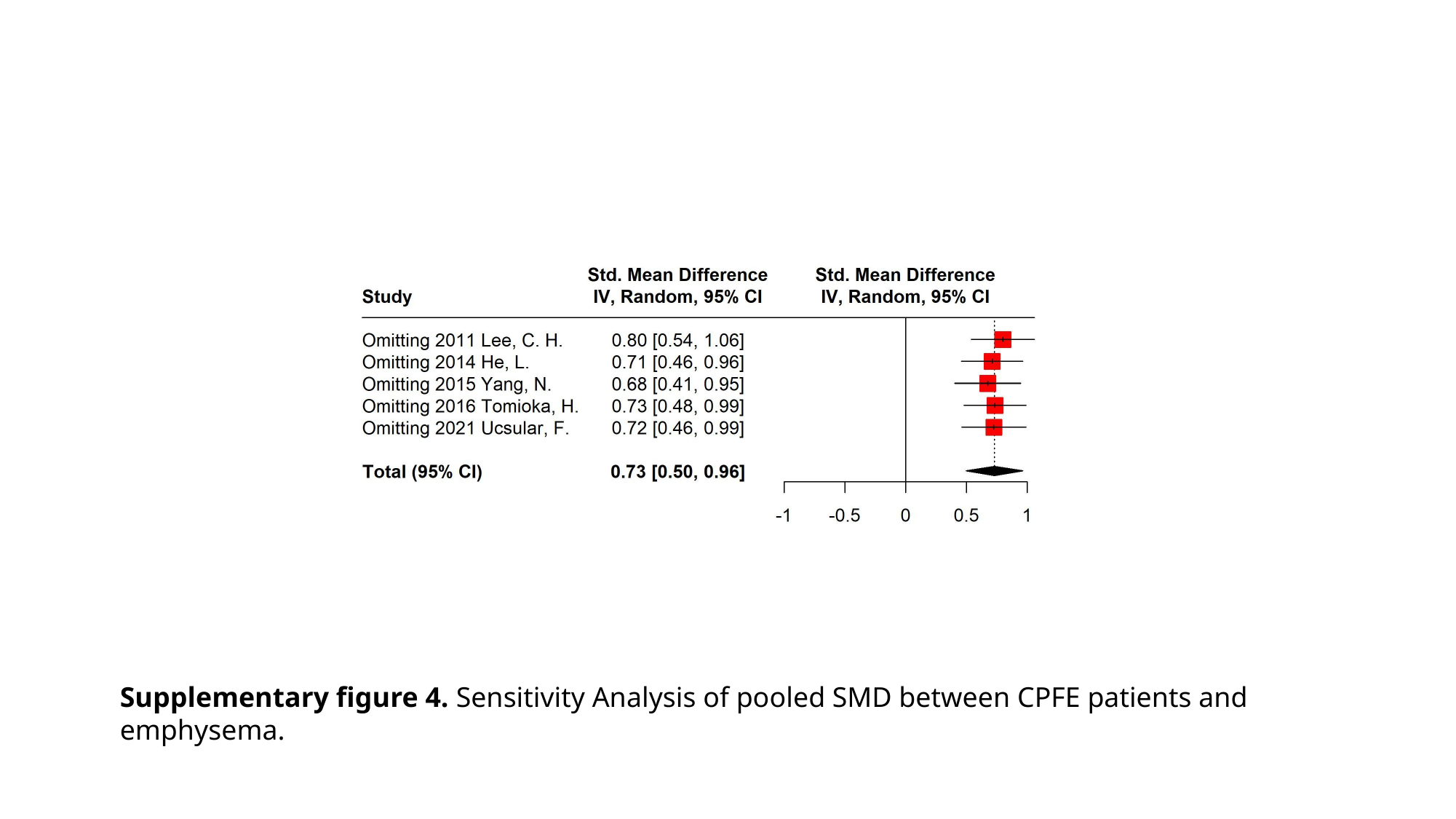

Supplementary figure 4. Sensitivity Analysis of pooled SMD between CPFE patients and emphysema.
